# Supplementary figures and images for: The histone modification regulator, SIN3, plays a role in the cellular response to changes in glycolytic flux
Source: PLoS One. 2025 Nov 26;20(11):e0335411. doi: 10.1371/journal.pone.0335411 (PMC12654916; doi:10.1371/journal.pone.0335411)

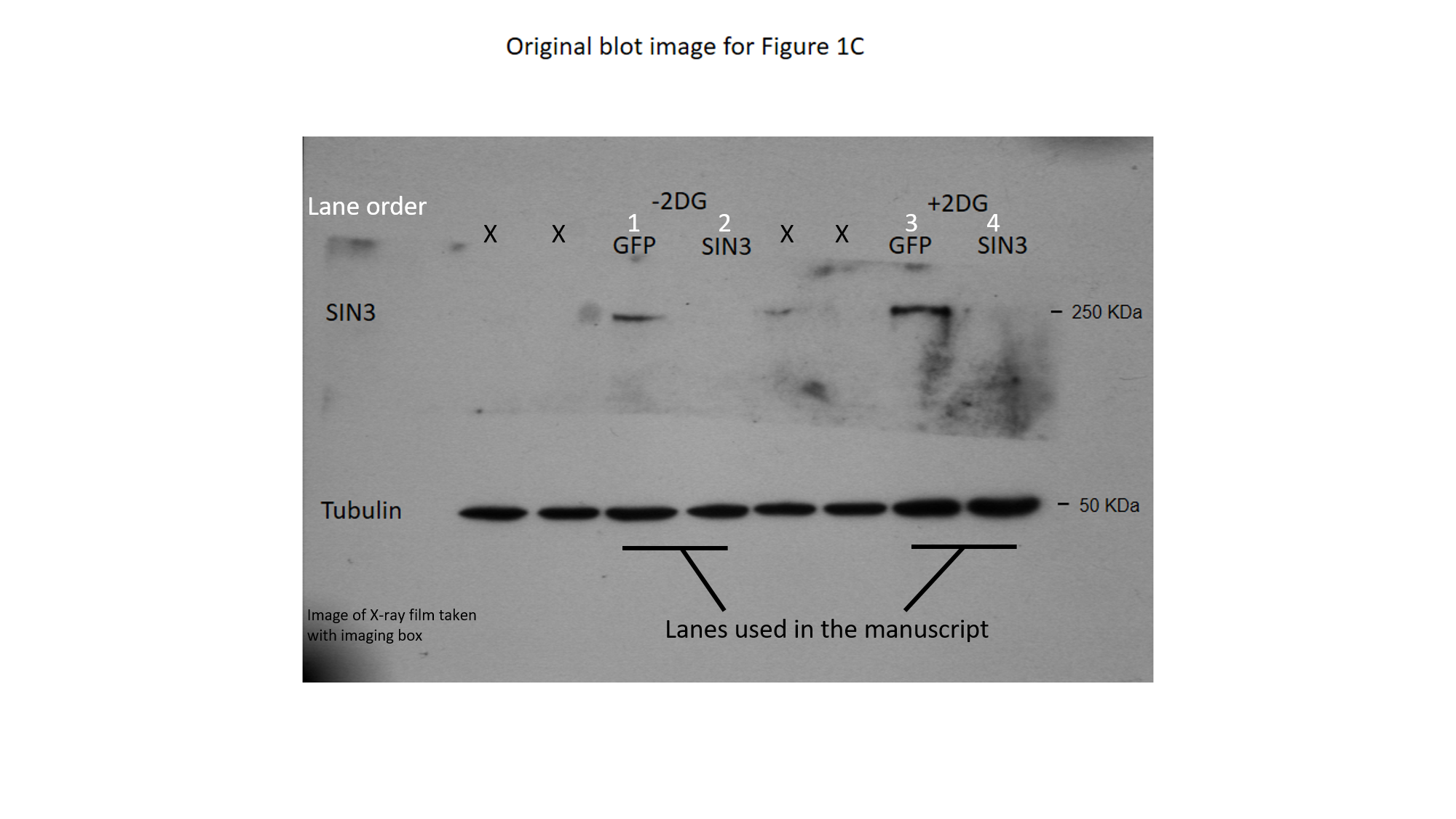

Supplement: S1 Raw Images — (TIF) [file pone.0335411.s001.tif]
